# Using data fusion with multiple imputation to correct for misclassification in self-reported exposure: a case-control study of cannabis use and homicide victimization

**DOI:** 10.1186/s40621-024-00545-x

**Published:** 2024-10-23

**Authors:** Seonghun Lee, Guohua Li, Stanford Chihuri, Yuanzhi Yu, Qixuan Chen

**Affiliations:** 1 Department of Biostatistics, Columbia University Mailman School of Public Health, New York, NY 10032 USA; 2 Department of Anesthesiology, Columbia University Vagelos College of Physicians and Surgeons, New York, NY 10032 USA; 3 Department of Epidemiology, Columbia University Mailman School of Public Health, New York, NY 10032 USA; 4 Division of Biostatistics, Department of Preventive Medicine, Northwestern University Feinberg School of Medicine, Chicago, IL 60611 USA; 5 Division of Social Solutions and Services Research, Nathan S. Kline Institute for Psychiatric Research, Orangeburg, NY 10962 USA

**Keywords:** Alcohol use, Cannabis use, Data integration, Missing at random, Multiple imputation, National surveys, Stratified bootstrapping

## Abstract

**Background:**

Cannabis use has been causally linked to violent behaviors in experimental and case studies, but its association with homicide victimization has not been rigorously assessed through epidemiologic research.

**Methods:**

We performed a case-control analysis using two national data systems. Cases were homicide victims from the National Violent Death Reporting System (NVDRS), and controls were participants from the National Survey on Drug Use and Health (NSDUH). While the NVDRS contained toxicological testing data on cannabis use, the NSDUH only collected self-reported data, and thus the potential misclassification in the self-reported data needed to be corrected. We took a data fusion approach by concatenating the NSDUH with a third data system, the National Roadside Survey of Alcohol and Drug Use by Drivers (NRS), which collected toxicological testing and self-reported data on cannabis use for drivers. The data fusion approach provided multiple imputations (MIs) of toxicological testing results on cannabis use for the participants in the NSDUH, which were then used in the case-control analysis. Bootstrap was used to obtain valid statistical inference.

**Results:**

The analyses revealed that cannabis use was associated with 3.55-fold (95% CI: 2.75–4.35) increased odds of homicide victimization. Alcohol use, being Black, male, aged 21–34 years, and having less than a high school education were also significantly associated with increased odds of homicide victimization.

**Conclusions:**

Cannabis use is a major risk factor for homicide victimization. The data fusion with MI method is useful in integrative data analysis for harmonizing measures between different data sources.

**Supplementary Information:**

The online version contains supplementary material available at 10.1186/s40621-024-00545-x.

## Background

Homicide has long been a major public health issue in the United States. It is a leading cause of death for those aged between 5 and 44 in 1980 and 2019 (Centers for Disease Control and Prevention 2022a). In 2020, homicide claimed 24,576 lives, yielding a death rate of 7.5 per 100,000 population (Centers for Disease Control and Prevention 2021a, 2022b). It is well known that excess alcohol consumption is positively associated with the risk of violence, including homicide, suicide, and sexual assault (Centers for Disease Control and Prevention 2022c; National Council on Alcoholism and Drug Dependence 2016). Almost one in four, or 2.7 million out of the 11.1 million victims of violent crime, report that the offender had been drinking alcohol prior to committing the crime each year (Greenfeld 1998). Of the 12,638 homicide victims with toxicological testing results in 9 states between 2004 and 2016, 37.5% tested positive for alcohol, 31.0% positive for cannabis, and 11.4% positive for both substances, and the prevalence of cannabis use detected in homicide victims increased from 22.3% in 2004 to 42.1% in 2016 (Nazarov and Li 2020).

Although cannabis use has been causally linked to violent behaviors in experimental studies (Allen et al. 1975; Alves and Carlini 1973; Alves et al. 1973; Beatty et al. 1984; Beezley et al. 1987) and case studies (Goldstein 1985), a more recent study calls into question a direct causal link between cannabis use and violent behaviors (De Perna et al. 2016). It is worth noting that findings from these experimental studies are not necessarily equally applicable to homicide perpetrators and homicide victims. The goal of this paper is to assess the association between cannabis use and homicide victimization, which has not been rigorously examined through epidemiologic research partly because toxicological testing data for the general population are lacking. In this paper, a case-control analysis aimed at assessing the association between cannabis use and the risk of homicide victimization was conducted using three national data systems, including the 2013–2014 National Violent Death Reporting System (NVDRS), the 2013–2014 National Survey on Drug Use and Health (NSDUH), and the 2013–2014 National Roadside Survey of Alcohol and Drug Use by Drivers (NRS). Cases were homicide victims recorded in the NVDRS and controls were participants in the NSDUH, a nationally representative sample. The exposure of primary interest was cannabis use, which was measured based on toxicological testing of blood specimens for the cases and self-report for the controls. Because self-reported cannabis use data might be more susceptible than toxicological testing to misclassification error, a case-control analysis directly comparing the NVDRS and NSDUH is neither feasible nor valid. In order to address this problem, we devised a data fusion with multiple imputation approach to correct for the misclassification error in the self-reported cannabis use data in the NSDUH by borrowing the data from the NRS, which contained both toxicological testing and self-reported data on cannabis use for a purposeful sample of drivers. To obtain valid statistical inference in the case-control analysis of the imputed NVDRS and NSDUH, we used stratified bootstrap inference with multiple imputation (von Hippel and Bartlett 2021; Yu et al. 2024).

## Methods

### Study population and data collection

*National Violent Death Reporting System*. The NVDRS is a population-based surveillance system that collects data from participating states in the US regarding violent deaths obtained from death certificates, coroner/medical examiner reports, law enforcement reports, and toxicology reports (Centers for Disease Control and Prevention 2021b). The 2013 NVDRS included data from 17 states (Alaska, Colorado, Georgia, Kentucky, Maryland, Massachusetts, North Carolina, New Jersey, New Mexico, Ohio, Oklahoma, Oregon, Rhode Island, South Carolina, Utah, Virginia, and Wisconsin), a total of 18,765 fatal incidents involving 19,251 deaths (Lyons et al. 2016). The reporting system included information about decedent demographic characteristics, whether alcohol and substance tests were positive, manner of death, and month in which the death occurred. In this study, the 2013 of 4,110 homicide victims aged 16 or older were included.

*National Survey on Drug Use and Health*. The NSDUH provides nationally representative survey data that contains information about the use of illicit drugs, alcohol, and tobacco among members of the US civilian, noninstitutionalized population aged 12 or older in all 50 states and the District of Columbia. Survey samples were selected using a stratified multistage sampling design and data were weighted to be representative of the US general population (United States Department of Health and Human Services. Substance Abuse and Mental Health Services Administration Center for Behavioral Health Statistics and Quality 2014). The survey included questions on respondent demographic characteristics, alcohol and substance use, and mental health. In this study, the 2013 data of 43,365 survey participants aged 16 or older were included.

*National Roadside Survey of Alcohol and Drug Use by Drivers*. The NRS is designed to gauge alcohol and drug use by drivers on the US roadways and has been conducted in 1973, 1986, 1996, 2007, and 2013, with non-alcohol drug data being included in the 2007 and 2013 surveys. Participants in the 2013 NRS were non-commercial drivers randomly selected at 300 locations across the 48 contiguous states during designated time segments (9:30 am to 11:30 am and 1:30 pm to 3:30 pm on Fridays and from 10 pm to midnight and 1 am to 3 am on both Friday and Saturday nights) (National Highway Traffic Safety Administration^ 2015^). The sample was selected using a multistage sampling method and survey weights were provided to make the sample representative of the U.S. driver population (National Highway Traffic Safety Administration 2017). The survey included questions on driver demographic characteristics, drinking and substance use, and trip and vehicle information. In addition, breath alcohol, oral fluid alcohol, and oral fluid drug concentration tests were administered and whole blood specimens were collected during the survey process. In this study, the 2013 data of 11,314 drivers aged 16 and older were included.

## Cannabis and alcohol use

In the NVDRS, alcohol and cannabis positivity were determined based on blood sample tests. Blood alcohol concentrations (BACs) were measured in grams per deciliter, and BACs of 0.01 g per deciliter or greater were considered alcohol positive (Center for Disease Control and Prevention 2022d). Cannabis blood tests provided binary results - whether the test for cannabis was positive or negative. Differently, self-reported alcohol and cannabis use were recorded as binary variables in the NSDUH, indicating whether one used alcohol or cannabis in the past month. Finally, alcohol and cannabis use were measured in both blood and oral fluid samples and asked with questionnaires in the NRS. Both the BACs and the raw alcohol levels in oral fluids were measured in milligrams per deciliter, and a value of 10 mg (or 0.01 g) per deciliter or greater was considered alcohol positive. Blood and oral cannabis tests indicated whether the test for Tetrahydrocannabinol was positive or negative. The questions about the last time of cannabis use (past 24 h, past 2 days, past month, over a month, beyond a year/never) and the frequency of weekly alcohol consumption (0, 1–2, 3–4, 5–7, 8–14, more than 14 drinks) were asked. We created the binary self-reported alcohol and cannabis use variables to be comparable with the self-reported variables in the NSDUH. Specifically, individuals who reported cannabis use in the past 24 h, past 2 days, or past month were categorized as cannabis use in the past month, and no use otherwise; and individuals who reported with non-zero alcohol consumption frequency were categorized as alcohol use in the past month, and no use otherwise.

## Covariates

The variables included as covariates in the case-control analysis are presented in Table 1. Demographic variables include age (16–20 years, 21–34 years, 35–49 years, 50–64 years, or $\:\ge\:$65 years), sex (male vs. female), race (White, Black, Hispanic, or Others), and education (less than high school, high school graduate, some college, or college graduate/some graduate). Categorizing the education variables was straightforward, as they were similarly defined across all three data sets. The age variable was continuous in the NVDRS and NRS but categorical in the NSDUH, so we adopted the NSDUH categories and collapsed some age groups. For race/ethnicity, despite slight differences in definitions across data sets, we classified participants as Hispanic, non-Hispanic White, non-Hispanic Black, and grouped all other non-Hispanic individuals as ‘Others’. We included these covariates because they are established risk factors for homicide victimization and potential confounders in the relationship between cannabis use and homicide victimization. All the covariates are fully measured in the NSDUH but have missing values in both the NRS and the NVDRS.

**Table 1:** Distributions of the baseline characteristics of the three US National Data Systems aged 16 years and older: drivers from the 2013-14 National Roadside Survey of Alcohol and Drug Use (NRS), US population from the 2013-14 National Survey on Drug Use and Health (NSDUH), homicide victims from the 2013 National Violent Death Reporting System (NVDRS)

Data source	NRS *n* = 11,314 Frequency (%)	NSDUH *n* = 43,465 Frequency (%)	NVDRS *n* = 4,110 Frequency (%)
**Age (years)**			
16–20	1,069 (11.7)	3,817 (8.8)	489 (11.9)
21–34	3,640 (39.8)	10,414 (24.0)	1,813 (44.1)
35–49	2,310 (25.2)	10,751 (24.7)	997 (24.3)
50–64	1,621 (17.7)	10,755 (24.7)	581 (14.1)
≥65	515 (5.6)	7,728 (17.8)	230 (5.6)
Missing	2,158	0	0
**Sex**			
Male	6,382 (58.3)	20,970 (48.2)	3,340 (81.3)
Female	4,566 (41.7)	22,495 (51.8)	770 (18.7)
Missing	365	0	0
**Race**			
White	4,952 (55.0)	28,458 (65.5)	1,229 (29.9)
Black	2,196 (24.4)	5,131 (11.8)	2,306 (56.1)
Hispanic	1,074 (11.9)	6,622 (15.2)	376 (9.1)
Others	776 (8.6)	3,254 (7.5)	199 (4.8)
Missing	2,316	0	0
**Education**			
Less than high school	715 (7.8)	5,616 (12.9)	887 (38.0)
High school graduate	2,115 (23.1)	12,441 (28.6)	1,055 (45.2)
Some college	3,222 (35.2)	11,365 (26.1)	248 (10.6)
College/some graduate	3,106 (33.9)	14,043 (32.3)	145 (6.2)
Missing	2,156	0	1,775
**Self-reported alcohol use**			
Yes	3,811 (58.4)	23,904 (55.0)	-
No	2,720 (41.6)	19,561 (45.0)	-
Missing	4,783	0	4,110
**Oral alcohol test**			
Positive	234 (2.8)	-	-
Negative	8,149 (97.2)	-	-
Missing	2,931	43,465	4,110
**Blood alcohol test**			
Positive	137 (2.7)	-	1,027 (44.7)
Negative	4,926 (97.3)	-	1,268 (55.3)
Missing	6,251	43,465	1,815
**Self-reported cannabis use**			
Yes	905 (11.7)	3,417 (7.9)	-
No	6,813 (88.3)	40,048 (92.1)	-
Missing	3,596	0	4,110
**Oral cannabis test**			
Positive	864 (10.3)	-	-
Negative	7,519 (89.7)	-	-
Missing	2,931	43,465	4,110
**Blood cannabis test**			
Positive	555 (11.0)	-	663 (46.8)
Negative	4,494 (89.0)	-	755 (53.2)
Missing	6,265	43,465	2,692

### Statistical analysis

We performed descriptive analysis to compare the distributions of the cannabis and alcohol use variables as well as the covariates between the three data systems. For the NRS and NSDUH, frequencies and the survey weighted percentage were provided; while for the NVDRS, frequencies and the unweighted percentage were reported. To assess the degree of misclassification associated with the self-reported results, we used the NRS data with complete pairs of self-reported use and blood test for both cannabis and alcohol to calculate the sensitivity and specificity of self-reported cannabis and alcohol use.

To correct for potential misclassification in the self-reported cannabis and alcohol use in the NSDUH, we devised a data fusion approach. We first homogenized the NSDUH and NRS data by ensuring both data sets had the same variables with consistent formats and definitions. We then concatenated the two data sets by stacking them, with one placed directly on top of the other. The oral and blood test results of cannabis and alcohol use in the NSDUH were treated as missing data. We then performed multiple imputation (MI) to fill in the missing test results in the NSDUH using the Chained Equations Multiple Imputation (CEMI) algorithm (Raghunathan et al. 2001; van Buuren 2007) assuming data are missing at random. The CEMI algorithm is an iterative procedure, imputing one variable at a time conditioning on all the other variables in the data set. To impute the missing binary test result of cannabis and alcohol use, we considered three different imputation models, including logistic regression, lasso logistic regression, and random forest. We compared the performance of the three imputation models using area under curve, sensitivity, and specificity with 10-fold cross-validation and found that the lasso logistic model yielded the highest area under curve (Supplementary eFigure 1). Hence, the lasso logistic regression was chosen as the imputation models in the imputation of test results of cannabis and alcohol use. The CEMI algorithm was implemented using the “mice” package (van Buuren et al. 2011) in R and created multiple imputations of blood test results of cannabis and alcohol use in the NSDUH, which were then used in the next step of the analyses.

There were missing values in both cannabis and alcohol blood test variables in the NVDRS. We multiply imputed the missing data in the NVDRS again using the “mice” package in R by assuming data are missing at random and created the same number of imputations as in the NSDUH. Then, each imputation of the NSDUH was concatenated with each imputation of the NVDRS by stacking them, with one placed directly on top of the other, and created combined imputed NSDUH and NVDRS data sets. For the purpose of modeling with the NSDUH data in the integrative data analysis, survey design variables were manually added to the NVDRS. Specifically, a unique value was assigned to each observation for the primary sampling unit variable; all observations were assigned with a single stratum value which is different from the strata values in the NSDUH; and the value 1 was assigned to each observation for the weight variable.

We fit weighted logistic regression models on the multiply imputed integrative data of the NSDUH and NVDRS, accounting for the stratified multistage sampling design in the NSDUH. In our regression models, homicide victimization is the outcome, cannabis use is the exposure, and demographics and alcohol use are the covariates. Because the NRS was involved in the imputation but was not used in the post-imputation case-control analysis, the conventional variance estimation method for multiply imputed data using the Rubin’s MI combining rules (Rubin 1987) does not apply anymore (Reiter 2008). Yu et al. (2024) showed that the bootstrapping with multiple imputation (BMI) yields valid statistical inference in this setting. The BMI method is implemented by first bootstrapping the samples and then conducting MI within each bootstrapped sample (von Hippel and Bartlett 2021). To obtain bootstrap samples, resampling was conducted on primary sampling units within each stratum for the NSDUH and NRS, and on homicide victims for the NVDRS. We conducted 200 bootstraps with 2 imputations within each bootstrapped combined sample. This yields 400 multiply imputed integrative data sets of the NSDUH and NVDRS. We fit one weighted logistic regression model on each imputed integrative data set. The results of 400 sets of regression coefficients were then pooled to obtain the final point estimates of the regression coefficients and bootstrap variance estimates that account for the variation between the multiple imputations within a bootstrapped data set, and between the bootstrapped data sets. The 95% confidence intervals (CI) of the regression coefficients are then obtained using a t-distribution with degrees of freedom computed by Satterthwaite approximation (von Hippel and Bartlett 2021). We called it model 1.

For comparison, we also fit the other three weighted logistic multivariable models. Model 2 was fitted using 20 multiply imputed NVDRS and NSDUH data after the process of data fusion with MI, with the variance of the regression coefficients estimated using the Rubin’s method for MI combination. Model 2 was included to show the difference in the 95% CI interval estimates using the Rubin’s method versus the BMI method. Models 3 and 4 considered a different case-control analysis using the participants in the NRS as the controls. Different from the NSDUH in which the participants represented the general U.S. population, the participants in the NRS only represented the drivers in the US. Therefore, the case-control analysis using the NRS as the controls is less desirable than that using the NSDUH as the controls. Because the blood test results of cannabis and alcohol use were available in the NRS, data fusion with MI was not required in the case-control analysis using the NRS as controls. Model 3 was fitted using the complete cases of the NVDRS and NRS data using listwise deletion of any incomplete observations. Model 4 was fitted using 20 multiply imputed NVDRS and NRS data, with the incomplete covariates imputed using the “mice” package in R by assuming data are missing at random and the variance of the regression coefficients estimated using the Rubin’s method for variance estimation.

## Results

Table 1 shows the distributions of the demographic variables as well as the cannabis and alcohol use variables in the NVDRS, NSDUH, and NRS. The 2013 NSDUH is a representative sample of the US general population, and the NRS is a representative sample of drivers only. Among individuals aged 16 years and older, compared to the US general population, drivers had higher percentage of males (58.3% vs. 48.2%), higher percentage of people aged between 21 and 34 (39.8% vs. 24.0%), lower percentage of White population (55.0% vs. 65.5%) but higher percentage of Black population (24.4% vs. 11.8%), higher percentage of some college or college education (69.1% vs. 58.4%), and higher percentage of self-reported cannabis use (11.7% vs. 7.9). On the other hand, there was much higher percentage of males (81.3%), Black people (56.1%), people with education less than or equal to high school (83.2%) among the homicide victims. It is also noticeable that 46.8% of the homicide victims had a positive blood cannabis test and 44.7% had a positive blood alcohol test; while only 11.0% and 2.7% of drivers had positive blood cannabis and alcohol test, respectively.

Table 2 shows the results of the sensitivity/specificity analyses for the self-reported cannabis and alcohol use with the blood test results as the gold standard using the complete pairs of self-reported and blood test results in the NRS. The self-reported cannabis use had a high specificity (94%) but low sensitivity (62%); whereas the self-reported alcohol use had a high sensitivity (88%) but low specificity (47%). This finding is expected because cannabis stays longer in the body than alcohol and the self-reported variables are based on the past month use. In addition, the sensitivity and specificity varied between different age, gender, race, and education subgroups. For example, the Black race group had lower sensitivity than the White race group in both the self-reported cannabis (56% vs. 66%) and alcohol (71% vs. 95%) use. These results indicate the existence of misclassification error in the self-reported cannabis and alcohol use variables and hence the correction for the misclassification is necessary. Further the misclassification is differential with the degree of misclassification related to demographic variables. These findings highlight the importance of correcting for misclassification error in the self-reported data in the NSDUH and the necessity in accounting for the covariates in the misclassification error adjustment.

**Table 2:** Sensitivity and specificity of self-reported cannabis and alcohol use from the NRS data sample with complete pairs of self-reported use and blood test for both cannabis and alcohol, stratified by age, sex, race, and education

	Cannabis use			Alcohol use		
	n	Sens (%)	Spec (%)	n	Sens (%)	Spec (%)
**Overall**						
All-inclusive	4,221	62	94	3,022	88	47
**Age (years)**						
16–20	439	66	88	235	100	58
21–34	1,762	63	91	1,441	86	40
35–49	1,039	62	98	744	88	53
50–64	755	44	98	480	91	52
≥65	226	100	99	122	100	52
**Sex**						
Male	2,361	66	94	1,714	90	40
Female	1,860	56	94	1,308	83	57
**Race**						
White	2,436	66	94	1,760	95	47
Black	931	56	94	670	71	46
Hispanic	434	70	96	301	90	53
Others	428	62	95	291	100	46
**Education**						
Less than high school	254	55	96	145	100	54
High school graduate	970	63	95	646	82	53
Some college	1,600	60	93	1,175	84	46
College/some graduate	1,397	67	95	1,056	95	45

The Supplementary eTable 1 repeated the analyses in Table 1 but showed the average estimates based on 20 imputations for each of the three data systems. The distributions of the demographic characteristics are like those in Table 1 using the complete cases. For NSDUH, the data fusion with MI estimated an oral alcohol positivity rate of 2.1% (compared to 2.7% among drivers), a blood alcohol positivity rate of 2.6% (compared to 3.0% among drivers), an oral cannabis positivity rate of 6.9% (compared to 9.8% among drivers), and a blood cannabis positivity rate of 7.4% (compared to 9.9% among drivers).

Table 3 shows the odds ratio (OR) and 95% confidence interval (CI) of the case-control analyses. According to model 1, cannabis use was associated with a 3.55-fold increase (95% CI: 2.75, 4.35) in the odds of homicide victimization. Alcohol use was also strongly associated with the homicide victimization with OR = 19.25 (95% CI: 12.25, 26.24), followed by being racially Black with OR = 5.27 (95% CI: 4.55, 6.0), being male with OR = 2.94 (95% CI: 2.59, 3.3), being aged 21 to 34 years old with OR = 1.79 (95% CI: 1.33, 2.25), and having education less than high school with OR = 1.68 (95% CI: 1.44, 1.91). Comparing model 1 to model 2, the point estimates of the regression coefficients are similar, but the BMI method used in model 1 yielded narrower confidence intervals for the coefficients of the drug variables than the Rubin’s method, because the latter overestimated the variance of the regression coefficients in this setting. Comparing model 1 to model 4, using drivers as the controls did not lead to much difference in the estimated associations between homicide victimization and the use of cannabis or alcohol, but there are notable differences in the ORs associated with the demographic variables. Finally, comparing model 3 to model 4, the MI led to a much larger analyzable data set, with the sample size increased by 3 times. Model 4 estimated a stronger association between homicide victimization and the cannabis and alcohol use and narrower confidence intervals than model 3. The comparison between models 3 and 4 highlights the limitations of complete-cases analysis, where listwise deletion of any incomplete observations could largely reduce statistical power and result in biased statistical inference. MI can be used to overcome these limitations.

**Table 3:** Odds ratio of homicide victimization in the case-control study with victims in the NVDRS as cases and general population in the NSDUH or drivers in the NRS as controls using four weighted logistic multivariable regression models: model 1 pools the 400 estimates of regression coefficients using 400 bootstrapped-then-imputed NVDRS and NSDUH data after data fusion; model 2 pools the 20 estimates of regression coefficients using 20 multiply-imputed NVDRS and NSDUH Data after data fusion; model 3 shows the estimates of regression coefficients using the complete cases of the NVDRS and NRS data using listwise deletion; model 4 pools the 20 estimates of regression coefficients using 20 multiply-imputed NVDRS and NRS

	NVDRS + NSDUH		NVDRS + NRS	
	Model 1 *n* = 47,582 OR (95% CI)	Model 2 *n* = 47,582 OR (95% CI)	Model 3 *n* = 5,205 OR (95% CI)	Model 4 *n* = 15,431 OR (95% CI)
**Age (years)**				
35–49	Ref	Ref	Ref	Ref
16–20	1.41 (1.08, 1.84)	1.37 (1.06, 1.78)	1.10 (0.67, 1.79)	0.51 (0.38, 0.69)
21–34	1.76 (1.38, 2.25)	1.84 (1.47, 2.29)	1.13 (0.79, 1.64)	0.81 (0.68, 0.95)
50–64	1.37 (1.08, 1.73)	1.46 (1.10, 1.94)	0.83 (0.56, 1.24)	0.79 (0.66, 0.96)
≥65	0.71 (0.54, 0.94)	0.70 (0.53, 0.93)	1.06 (0.51, 2.19)	1.48 (1.05, 2.09)
**Sex**				
Female	Ref	Ref	Ref	Ref
Male	2.94 (2.60, 3.31)	2.91 (2.59, 3.27)	2.19 (1.65, 2.91)	1.73 (1.45, 2.07)
**Race**				
White	Ref	Ref	Ref	Ref
Black	5.25 (4.58, 6.02)	5.19 (4.51, 5.97)	2.56 (1.32, 4.96)	2.61 (1.60, 4.27)
Hispanic	0.73 (0.61, 0.87)	0.72 (0.59, 0.87)	2.59 (1.51, 4.42)	0.88 (0.59, 1.30)
Others	1.59 (1.31, 1.93)	1.49 (1.15, 1.93)	1.84 (1.03, 3.28)	1.19 (0.78, 1.80)
**Education**				
High school graduate	Ref	Ref	Ref	Ref
Less than high school	1.67 (1.45, 1.92)	1.69 (1.45, 1.97)	2.23 (1.68, 2.97)	2.47 (1.97, 3.09)
Some college	0.32 (0.26, 0.38)	0.31 (0.26, 0.38)	0.19 (0.14, 0.26)	0.20 (0.17, 0.25)
College/some graduate	0.19 (0.16, 0.23)	0.19 (0.16, 0.24)	0.18 (0.12, 0.27)	0.14 (0.11, 0.19)
**Drug**				
Alcohol (positive vs. negative)	18.53 (12.64, 27.16)	19.09 (11.51, 31.65)	15.83 (9.89, 25.33)	17.99 (12.87, 25.15)
Cannabis (positive vs. negative)	3.50 (2.80, 4.39)	3.59 (2.77, 4.64)	2.58 (1.67, 3.97)	3.53 (2.60, 4.79)

## Discussion

This study is among the first to assess the association between cannabis use and homicide victimization using rigorous statistical analytic inference and multiple national data systems. Because the data source for cases (i.e., the NVDRS) contained toxicological testing results for cannabis and alcohol use but the data source for controls (i.e., the NSDUH) collected self-reported data only, it is impossible to perform a valid case-control analysis without the employment of the data fusion with multiple imputation method. The data fusion with multiple imputation method we proposed in this paper has wide applications and could facilitate rigorous research using existing data systems to address challenging and important questions.

In this population-based case-control study, we demonstrate that there is a strong association between cannabis use and homicide victimization. The finding that cannabis use is associated with increased odds of being homicide victim is consistent with previous studies (Hohl et al. 2017; Darke et al. 2019; Nazarov and Li 2022). Additionally, the estimated associations of alcohol use and demographic characteristics with homicide victimization obtained by the data fusion with MI method are in line with other reports (Darke et al. 2019). This indicates that data fusion with multiple imputation is useful for integrative data analysis in the context of misclassification and missing values. It is interesting to find that the association between cannabis use and homicide victimization was similar between the analyses using the NSDUH as the controls and using the NRS as the controls, even though one represented the US general population and the other represented the driver populations.

Because the data used for imputations (NRS + NSDUH) in the data fusion step differ from the data used in the post-imputation analyses (NVDRS + NSDUH), Rubin’s method for MI variance estimation may not yield valid statistical inference and no longer be applicable. We applied the bootstrapping with multiple imputation method for the variance estimation instead. Our analysis shows that the 95% CIs of the regression coefficients estimated using the bootstrapping with MI method are narrower than those estimated using the Rubin’s method applied to MI only. This result agrees with the findings in other studies (Yu et al. 2024).

Although the NRS and the NSDUH samples differ in demographics, our data fusion approach assumes that the conditional distributions of blood cannabis and alcohol use are similar between the two populations when controlling for the same demographic factors. To assess the validity of this assumption, we analyzed the observed self-reported cannabis and alcohol use data as outcomes and found that the regression coefficient estimates of each covariate in weighted logistic regression are similar between the two data sets. This suggests that the conditional distributions of self-reported cannabis and alcohol use, given covariates values, are comparable between the NSDUH and the NRS, despite some discrepancies in their marginal distributions.

The study has some limitations. The questions about cannabis and alcohol use in the NRS were not framed exactly same as in the NSDUH. Specifically, cannabis use was asked about the last time of use (past 24 h, past 2 days, past month, over a month, beyond a year/never) in the NRS but a binary variable indicating whether one used cannabis in the past month was asked in the NSDUH. For alcohol use, the frequency of weekly alcohol consumption was asked in the NRS but a binary variable indicating whether one used alcohol in the past month was recorded in the NSDUH. We created binary variables of cannabis and alcohol use from the questions asked in the NRS to make the self-reported cannabis and alcohol use variables comparable between the two data systems, but this is less ideal than if the questions were framed the same. Additionally, the self-reported bias may differ between responses collected on ‘Roadside’ in the NRS and those at ‘home’ in the NSDUH. However, there is no known literature indicating difference between these settings. If a difference does exist, under-reporting is more likely to occur on roadside than at home, which would make our findings conservative. Another limitation is that the cases are from only 17 states in the NVDRS, while the control data include all states due to unavailability of state-specific information in the publicly available NSDUH data. However, the homicide death rate in these 17 states was similar to the national rate (4.13 vs. 4.52 per 100,000 population in 2013), and these 17 states accounted for 28.7% of U.S. homicide cases in 2013. Therefore, we believe our findings can still be generalized to the entire country. Further, we assume that the data are missing at random (MAR) given all the variables included in the imputation models. These variables are chosen because they are well known risk factors associated with homicide and cannabis and alcohol use. However, unmeasured confounders, such as psychiatric conditions, history of substance abuse, and depression symptoms, may still be present. The last thing to note is that the computation involved in the bootstrapping with MI is intensive. In this study, we have tried 200 bootstraps with 2 imputations, 200 bootstraps with 3 imputations, and 300 bootstraps with 3 imputations. There was little difference in the estimates. Therefore, we recommend a combination of 200 bootstraps with 2 imputations per bootstrap, which was also recommended by the literature (von Hippel and Bartlett 2021).

Nevertheless, the data fusion with MI method described in this study appears to be a powerful tool for integrative data analysis in epidemiologic studies. Its capacity to harness and harmonize data on variables across multiple data sources could vastly bolster the utility of individual data systems and open new horizons for epidemiologists. Our application of this novel method to three national data systems reveals a robust association between cannabis use and significantly increased risk of homicide victimization while reaffirming the well-documented role of alcohol use and demographic factors in homicide victimization.

## Conclusions

Cannabis use is a major risk factor for homicide victimization. Additionally, alcohol use, being Black, male, aged 21–34 years, and having less than a high school education were significantly associated with increased odds of homicide victimization. The data fusion with multiple imputation method is a powerful tool for harmonizing measures between different data sources in integrative data analysis. For valid statistical inference, variance estimation should be performed using the bootstrapping with multiple imputation method.

## Electronic supplementary material

Supplementary Material 1

## Data Availability

The data are not available for replication because the NVDRS and NRS are not publicly available. However, the computing codes are available in the project GitHub: https://github.com/Huneel7/Cannabis-Homicide-Code.

## Abbreviations

NVDRS: National Violent Death Reporting System
NSDUH: National Survey on Drug Use and Health
NRS: National Roadside Survey of Alcohol and Drug Use by Drivers
BAC: Blood alcohol concentration
MI: Multiple imputation
BMI: Bootstrapping with multiple imputation
CEMI: Chained equations multiple imputation
OR: Odds ratio
CI: Confidence interval
