# Supplementary material for: Using data fusion with multiple imputation to correct for misclassification in self-reported exposure: a case-control study of cannabis use and homicide victimization
Source: Inj Epidemiol. 2024 Oct 23;11:57. doi: 10.1186/s40621-024-00545-x (PMC11515600; doi:10.1186/s40621-024-00545-x)
Supplement: Supplementary file 1 — Supplementary Material 1 [file 40621_2024_545_MOESM1_ESM.docx]

SUPPLEMENT DIGITAL CONTENT

**eTable 1**. Weighted Distribution of the Baseline Characteristics of Three US National Data Samples Aged 16 Years and Older: Drivers from the 2013-14 NRS, US General Population from the 2013-14 NSDUH, Homicide Victims from the 2013 NVDRS, by Averaging 20 Imputations for Each Sample.

| Data Source | NRS | NSDUH | NVDRS |
| --- | --- | --- | --- |
|  | n = 11,314 | n = 43,465 | n = 4,110 |
|  | Frequency (%) | Frequency (%) | Frequency (%) |
| **Age (years)** |  |  |  |
| 16-20 | 1,487 (13.1) | 3,817 (8.8) | 489 (11.9) |
| 21-34 | 4,439 (39.2) | 10,414 (24.0) | 1,813 (44.1) |
| 35-49 | 2,771 (24.5) | 10,751 (24.7) | 997 (24.3) |
| 50-64 | 1,926 (17.0) | 10,755 (24.7) | 581 (14.1) |
| ≥65 | 691 (6.1) | 7,728 (17.8) | 230 (5.6) |
| **Sex** |  |  |  |
| Male | 6,566 (58.0) | 20,970 (48.2) | 3,340 (81.3) |
| Female | 4,748 (42.0) | 22,495 (51.8) | 770 (18.7) |
| **Race** |  |  |  |
| White | 6,337 (56.0) | 28,458 (65.5) | 1,229 (29.9) |
| Black | 2,528 (22.3) | 5,131 (11.8) | 2,306 (56.1) |
| Hispanic | 1,464 (12.9) | 6,622 (15.2) | 376 (9.1) |
| Others | 985 (8.7) | 3,254 (7.5) | 199 (4.8) |
| **Education** |  |  |  |
| Less than high school | 1,001 (8.8) | 5,616 (12.9) | 1,554 (37.8) |
| High school graduate | 2,736 (24.2) | 12,441 (28.6) | 1,839 (44.8) |
| Some college | 3,785 (33.5) | 11,365 (26.1) | 448 (10.9) |
| College/Some graduate | 3,792 (33.5) | 14,043 (32.3) | 268 (6.5) |
| **Self-reported alcohol use** |  |  |  |
| Positive | 6,298 (55.7) | 23,904 (55.0) | - |
| Negative | 5,016 (44.3) | 19,561 (45.0) | - |
| **Oral alcohol test** |  |  |  |
| Positive | 308 (2.7) | 922 (2.1) | - |
| Negative | 11,006 (97.3) | 42,543 (97.9) | - |
| **Blood alcohol test** |  |  |  |
| Positive | 345 (3.0) | 1,149 (2.6) | 1,825 (44.4) |
| Negative | 10,969 (97.0) | 42,316 (97.4) | 2,285 (55.6) |
| **Self-reported cannabis use** |  |  |  |
| Positive | 1,303 (11.5) | 3,417 (7.9) | - |
| Negative | 10,011 (88.5) | 40,048 (92.1) | - |
| **Oral cannabis test** |  |  |  |
| Positive | 1,112 (9.8) | 2,988 (6.9) | - |
| Negative | 10,203 (90.2) | 40,477 (93.1) | - |
| **Blood cannabis test** |  |  |  |
| Positive | 1,125 (9.9) | 3,197 (7.4) | 1,873 (45.6) |
| Negative | 10,189 (90.1) | 40,268 (92.6) | 2,237 (54.4) |

**eFigure 1.** Comparison of Three Imputation Models for the Cannabis and Alcohol Blood Test Variables Using 10-fold Cross-Validation.
